# Analysis of Actin FLAP Dynamics in the Leading Lamella

**DOI:** 10.1371/journal.pone.0010082

**Published:** 2010-04-15

**Authors:** Igor R. Kuznetsov, Marc Herant, Micah Dembo

**Affiliations:** Biomedical Engineering, Boston University, Boston, Massachusetts, United States of America; University of Milano-Bicocca, Italy

## Abstract

**Background:**

The transport of labeled G-actin from the mid-lamella region to the leading edge in a highly motile malignant rat fibroblast line has been studied using fluorescence localization after photobleaching or FLAP, and the transit times recorded in these experiments were so fast that simple diffusion was deemed an insufficient explanation (see Zicha et al., Science, v. 300, pp. 142–145 [1]).

**Methodology/Principal Findings:**

We re-examine the Zicha FLAP experiments using a two-phase reactive interpenetrating flow formalism to model the cytoplasm and the transport dynamics of bleached and unbleached actin. By allowing an improved treatment of effects related to the retrograde flow of the cytoskeleton and of the geometry and finite thickness of the lamella, this new analysis reveals a mechanism that can realistically explain the timing and the amplitude of all the FLAP signals observed in [1] without invoking special transport modalities.

**Conclusions/Significance:**

We conclude that simple diffusion is sufficent to explain the observed transport rates, and that variations in the transport of labeled actin through the lamella are minor and not likely to be the cause of the observed physiological variations among different segments of the leading edge. We find that such variations in labeling can easily arise from differences and changes in the microscopic actin dynamics inside the edge compartment, and that the key dynamical parameter in this regard is the so-called “dilatation rate” (the velocity of cytoskeletal retrograde flow divided by a characteristic dimension of the edge compartment where rapid polymerization occurs). If our dilatation hypothesis is correct, the transient kinetics of bleached actin relocalization constitute a novel and very sensitive method for probing the cytoskeletal dynamics in leading edge micro-environments which are otherwise very difficult to directly interrogate.

## Introduction

Fluorescence localization after photobleaching (FLAP) is a technique developed in the laboratory of Graham Dunn, whereby the proteins present in a localized region of the cytoplasm are photo-labeled and then tracked to ascertain their subsequent transport and fate [2], [3]. The investigations we report here were motivated by experiments of Zicha and coworkers, applying this technique to the dynamics of the actin cytoskeleton in the leading lamella of T15 cells, a line of transformed rat fibroblasts [1]. To carry out these studies, actin monomers labeled with yellow fluorophore and monomers labeled with cyan fluorophore were introduced into the cytoplasm via dual transfection with appropriate DNA constructs. Cells were then grown until a steady state of uniform cytoskeletal labeling was achieved. Subsequently, one of the dyes was photobleached for a few seconds in a narrow strip centered some microns behind the leading edge and extending transversely across the lamella. The relative prevalence of bleached and total actin in various cellular compartments was then studied as a function of time.

Zicha and coworkers concluded that, in general, a good many of the actin monomers from the bleach zone are very rapidly transported to the leading edge of the cell where they then become concentrated, particularly along segments of the edge undergoing fast protrusion. For example, in some measurements, the intensity of the measured FLAP signal implied that about 40% of the total actin present at a protruding edge was derived from monomer that 2 seconds previously had been situated within a bleaching zone more than ten microns away. This raises the question of what mechanism(s) can explain such profuse, rapid, and seemingly targeted movements of G-actin. To address this issue, Zicha and coworkers undertook extensive modeling efforts which finally led them to conclude that diffusion alone is not sufficient, and that some sort of active transport is needed.

To supplement diffusion, Zicha at al. initially considered the possibility of molecular motors somehow towing actin as cargo. However, the possibility that these motors were of the known classes associated with microtubules was discounted based on the paltry negative effects produced by specific inhibitors of such motors. Similar studies with inhibitors of the various myosin I motors also ruled out towing by members of this class. In the case of myosin II, a strong effect of specific inhibitors on the FLAP signal was noticed, but this class of myosin is generally assumed to be associated with muscle-like contraction and pressure-driven cytosolic flow, and has seldom if ever been implicated in the towing of specific cytoplasmic cargoes.

In view of their control studies, Zicha and coworkers concluded that G-actin transport aided by pressure-driven solvent flow through channels in the F-actin gel was the most likely explanation of the data. This *advection hypothesis* is controversial but it seems plausible in view of the fact that it requires only contraction mediated by myosin II, and in view of the lack of available alternatives. The idea also has some precedent in certain primitive motile systems. For example, the fountain flow in *Amoebae Proteus* [4] and the shuttle streaming of *Physarum polycephalum* [5] are clear cases where rapid cytosolic flows are driven by pressure gradients caused by the activity of myosin II. In addition, Keller et al. [6] have described a form of motility in a line of bladder carcinoma cells which involves formation of a large persistent bleb at the advancing margin. Such blebs (usually transient) are also seen near the leading edge in other cell types and are due to myosin-generated pressure gradients and associated hydraulic flows [7].

In what follows, we re-examine the theoretical arguments indicating that diffusion is too slow to explain the reported actin dynamics in T15 cells, and we ask if there is some other mechanism, not involving specialized transport, that could explain the FLAP data. We approach these issues by constructing a computer model of mass transport and FLAP dynamics in a leading lamella that is sufficently detailed to include processes such as channel flow through a porous cytoskeleton as suggested by Zicha et al. Based on simulations, we find that this model does indeed produce results that are quite close to what was observed by Zicha et al., but that the reasons for this success have nothing to do with channel flow or with special transport of any kind. To the contrary, the simulations suggest a completely different explanation for the enhanced FLAP signals at the protruding boundaries and for the modulation of FLAP signals by myosin. We call this conjecture the *dilatation hypothesis*.

## Methods

### Computational Domain for the T15 Fibroblast

**Figure 1:**
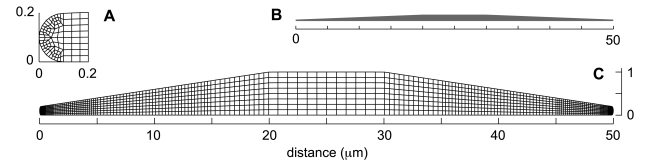
Computational mesh and geometry. (A) Leading edge compartment. (B) True geometry of the computational domain. The cell is represented in a sagittal cross-section; 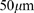 in length, 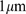 in height, and 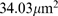 in area. (C) Computational mesh detail. The vertical axis is stretched for visibility.

On the timescale of a typical FLAP experiment (i.e. several tens of seconds), the background state of the cell and its cytoskeleton are usually changing slowly compared to the redistribution dynamics of the newly bleached material. We therefore model the sagittal cross-section of an adherent T15 cell by a closed two-dimensional computational domain with a fixed and prespecified geometry that is idealized and simplified and yet conforms with available morphometric data (see Fig. 1). Our simplified domain involves four geometric parameters. The first of these is the total length of the cell from tip to tail, 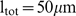. The second and the third are the length (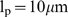) and the thickness (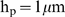) of a central “body” or plateau zone where the cell attains its maximum thickness and where the nucleus is usually found. Between the central plateau and the left and right edges, the thickness of the domain tapers down via linear ramps. These ramps can be viewed as representing the leading lamella and the tail regions in the case of a polarized crawling cell moving to left or right or else the two halves of a symmetric stationary cell with typical “fried egg” morphology. Finally, at the front and the back (or left and right), the dorsal and the ventral surfaces of our domain are joined by small semi-circular caps (see insert in Fig. 1). The cap radius, 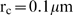, is the fourth and final geometric parameter.

We should note that as with all biological quantities, the precise values of the geometric parameters of Table 1 vary from cell to cell and that the numbers we have given are representative only of an average case. They are derived not only from the micrographs provided by Zicha et al. but also from a survey of published images of similar rat fibroblasts [8]–[11].

**Table 1:** Parameters used.

Parameter	Symbol	Units	Value	
Cap radius	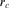	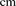	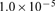	a
Plateau thickness	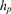	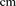	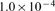	b
Total length	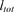	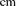	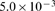	
Plateau length	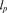	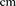	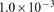	
Average actin volume fraction	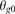	–	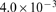	c
F/G ratio (caps)	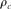	–	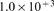	
F/G ratio (bulk)	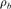	–	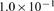	d
F-actin lifetime	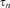	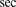	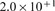	d
G-actin diffusion coefficient	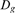	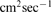	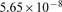	d
Specific network viscosity	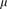	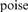	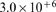	e
Specific network swelling	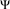	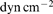	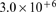	f
Network-solvent drag	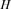	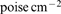	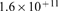	f

^*a*^approximates the leading edge thickness of the lamella reported in [8], [9].

^*b*^corresponds to the value reported in [10] for well-spread fibroblasts.

^*c*^calculated based on the value of 120 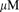 [9], [27].

^*d*^value reported in [1].

^*e*^determined via micropipette aspiration of human neutrophil [17].

^*f*^estimate from [17].

### Cytoplasmic Field Equations

We now suppose that the interior of our geometric model is populated by some distribution of actin filaments which form a kind of a weakly cross-linked spongy mass that we call the network phase, or simply the *network*. The pores of our network material are presumed to be filled with an aqueous medium, or *cytosol*, so that the overall composite of the two phases is incompressible. The boundaries (or surfaces) of the model domain are assumed to be impermeable to flow of both cytosol and network. We also assume that the ventral surface of the cell is attached to the substratum by transmembrane adhesion proteins which are sufficient to promote strong binding and anchorage of the network on this surface. On the dorsal surface of the domain and on the end caps, there should also be anchorage of the network to the membrane. However, since the lipid membrane is fluid, these anchorage sites are able to slip tangentially to the domain boundary.

To cast these general thoughts into precise equations, we will make use of the Reactive Interpenetrating Flow (RIF) formalism [12], [13]. This is a well studied approach to modeling of the cytoplasm, similar in concept to a recently proposed “poro-elastic” model [14]. Although the RIF method has not previously been used to model photobleaching or FLAP experiments, there is no difficulty with this sort of application and it has the advantage of enabling access to a large catalog of efficient and reliable software that has been successfully applied for modeling other cytomechanical processes. These include for example, cytokinesis in the sea urchin egg [15], micropipette aspiration of passive neutrophils [16], and neutrophil crawling and pseudopod protrusion [17], neutrophil phagocytosis [18], [19]. Below, we write down the mass transport equations needed for the current 2D sagittal cross section model of a T15 cell. We then consider the additional factors involved in specifying the velocity fields of the cytoplasm and cytosol and for calculating the outcome of FLAP experiments. We omit the details of the numerical methodology since this has been discussed previously in the various sources cited above.

#### Mass Transport

In the usual RIF notation we let network- and cytosol-associated quantities be indicated by the subscripts 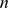 and 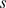, respectively. Thus, 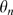 and 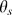 are the volume fractions of the two phases, while 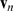 and 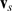 stand for associated velocity vectors. Neglecting the possibility of void volume or of a third phase, the sum of the two volume fractions must be unity everywhere in the computational domain. In other words, 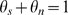, so only one volume fraction needs to be calculated.

The change in the local network concentration is governed by a continuity equation
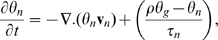
(1)where the term in parentheses represents the net rate of actin polymerization. The coefficient 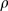 represents the ratio of F- over G-actin volume fractions at chemical equilibrium, and 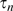 is the characteristic lifetime of an actin monomer in the filamentous pool. In principle, both chemical parameters can be functions of hidden variables that impart a dependency on position and time.

The evolution of G-actin is also governed by a continuity equation. However, in this case convective transport is governed by the solvent phase velocity, there is a diffusion term, and the reaction has the opposite sign. This yields
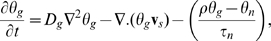
(2)where 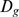 is the G-actin diffusion constant. It is worth noting that 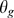 is measured in terms of the volume fraction, which is some sub-fraction of the solvent phase. This means that, strictly speaking, the effective concentration of G-actin with respect to the solvent is given by the ratio 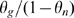. One would generally expect the diffusion constant of a material dissolved in the solvent to be proportional to the solvent volume fraction (see [20]) but we neglect this in writing Eq. 2 under the general presumption that 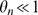.

Since the FLAP technique is concerned only with the mass transport of G and F actin, in principle we could now completely disregard the cytoskeletal physics and simply take the functions 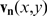 and 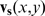 as “black-box” input parameters, to be specified empirically or in accordance with measurements. In this case, Eqs. 1 and 2 would stand on their own, and our model description would be complete. This purely “kinematic” approach has some superficial attractions but it lacks elegance and neglects the fact that we are not really completely free to specify the two phase velocities in an arbitrary fashion. For example, we know that the solvent is ultimately a passive material that can move only if driven by motions of the network. Thus, except possibly at a few isolated points in the computational domain, it is difficult to see any physical basis for setting 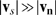. Additionally, it is evident that the overall cytoplasm is an incompressible composite mixture which implies that the divergence of the net cytoplasmic volume flux must vanish on a point-wise basis:
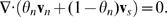
(3)Obviously, a completely ad-hock kinematic approach to specifing the values for 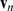 and 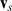 fails to take account of such basic realities.

#### Momentum Conservation

Since the purely kinematic approach has serious drawbacks, we propose instead to determine the functions 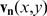 and 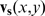 implicitly via a “toy” or “quasi-dynamical” model for momentum balance that is simplified but still incorporates enough physics to be plausible. There is no harm or loss of generality in this approach, provided one remembers that the model is nothing but a device or deus ex machina for generating internally consistent velocity fields.

Accordingly, we write the force balance for the solvent phase as
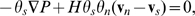
(4)where the first term gives the force acting per unit volume of solvent due to gradients of hydrodynamic pressure 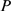, and the second is the force due to inter-phase friction (i.e. Darcy drag).

We next write the force balance on the network phase in a similar fashion. The only difference is that in this case we must include terms for swelling and contractile forces that tend to expand or shrink the network, and terms for viscous stresses that tend to resist gradients of network velocity. Adding all this up yields
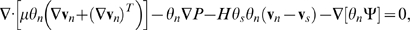
(5)where 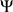 (the swelling coefficient) is a measure of the difference between the repulsive and attractive (or contractile) forces acting on the network filaments, and the parameter 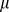 is a measure of the network shear viscosity.

By judicious choice of the boundary conditions and of the coefficients 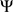, 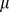, and 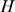, solutions of Eqs. 3, 4, and 5 allow easy control of the cytosol velocity and the network velocity in our computational domain in a way that also satisfies the minimal physical constraints mentioned previously. Furthermore, the control coefficients provide an intuitive contact with the hidden molecular factors which underlie the cytoskeletal behavior. For example, 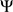 represents the sum of competing repulsive and attractive interactions distilled and condensed into a single scalar quantity. This is positive in the case where inter-filament repulsions are dominant and it is therefore usually called the “swelling” stress [17]. In molecular terms, the repulsions could be due to entropic and electrostatic forces between filaments, whereas the filament-filament attractive forces could be mediated by the actions of myosin II molecules. In a similar spirit, the coefficient 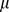 describes a very simple rheological model of the cytoskeleton wherein entanglements and cross-links between filaments are mediated by weak non-covalent bonds which have only a transient lifetime. Stress-induced fracture and rearrangement of these bonds then means that the network phase behaves like a viscous fluid that eventually flows and relaxes under the action of shearing stresses. The value of the viscosity coefficient will be large when the bonds are numerous, or when they resist rupture, or when the filament length is large. Finally, various standard treatments on the molecular or structural origins for the coefficient of Darcy's law, 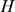, can be found in text books on flow in porous media. There it is shown that this parameter depends on the solvent viscosity and on the diameter, orientation, length, and density of the actin filaments (see citations in Table 1).

#### Initial and Boundary Conditions

For consistency with the nomenclature used by Zicha et al., we take the zero of time to be at the end of the bleaching interval. For purposes of modeling, this is assumed to be sufficiently long after the true starting condition, so that a steady state distribution of the F- and G-actin pools has developed. Accordingly, at the true computational starting point (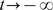), it is enough to say that all the actin of the cell is in the G form and is uniformly distributed with some specified volume fraction 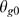. As already mentioned, all boundaries of the computational domain are assumed to be impermeable. In addition, the network velocity is assumed to satisfy “stick” boundary conditions on the ventral surface of the computational domain and “free slip” on all other surfaces. Because of Eq. 4, the condition of boundary impermeability translates into a requirement that the normal derivative of the pressure field should be zero.

### Modeling of FLAP Measurements

In the FLAP experiments of Zicha et al., the T15 cells were simultaneously transfected with two genes for 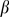-actin, one copy fused to yellow fluorescent protein (YFP) and the second copy fused to cyan fluorescent protein (CFP). At the start of an experiment, the YFP and CFP signals were recorded over the whole cell, and the two signals were uniformly rescaled so as to be identical in this reference state. This normalization compensates for any differences in labeling efficiency, quantum yield, absorbance, and so forth. Essentially, after this processing, the two fluorescence signals are logically equivalent to the signals that would be observed if all molecules of actin in the cell were doubly labeled with equal amounts of CFP and YFP (see [2] for further details on why this is so). For the sake of simplicity, we will henceforth work as if this ideal limit always holds.

Since both the YFP and CFP signals co-localize uniformly with the actin monomer in all states, in any given pixel, prior to bleaching, each signal will be proportional to the integral of the total actin density through the thickness of the lamella,
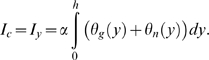
Here 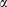 is the constant of proportionality, 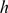 is the height of the lamella at the pixel location, and the integral extends over the lamella thickness. We may also write this equation in the equivalent form
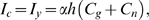
(6)where the upper case 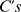, with appropriate subscripts, are used to denote the thickness averages of 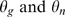.

To start a FLAP experiment, one of the fluorophores (YFP) is photobleached in a particular cell region (subsequently called the bleach zone). For the experiments of Zicha et al., this region was typically a strip about 3 microns wide extending across the lamella and centered 5–20 microns behind the leading edge. In order to calculate the results of a FLAP experiment using the definitions above, we need to introduce additional transport equations to follow the production, reaction, and transport of bleached actin in the G and F states, 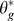 and 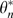. Due to the linear character of our chemical reaction terms (see above), the continuity equations for these bleached species are exactly the same as for the unbleached equivalents, Eqs. 1 and 2.

Since the CFP channel is not affected by the bleaching, the signal from this species still satisfies Eq. 6. However, as soon as some of the YFP molecules are bleached, the signal from this fluorophore is given by
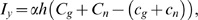
(7)where the lower case 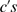 denote thickness averages of those actin monomers with bleached YFP. The *absolute* FLAP signal at a given pixel of an image is then calculated as the difference between the normalized CFP and YFP intensities. In view of the results just given, this is 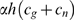.

The FLAP *ratio* within each pixel is calculated by taking the local absolute FLAP signal and dividing it by the normalized signal of the reference fluorophore (CFP). It can be seen that the resulting quantity,
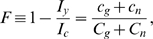
(8)is proportional only to the *fraction* of total actin in the lamella cross-section that is bleached (see supplement materials to [1], page 3). The main advantage of the FLAP ratio is that it represents a purely intensive property of the cytoplasm. It does not depend on the thickness of the lamella, or on the absolute density of actin monomers in the cross section, or on the percentage of actin monomers that are labeled, or on quantities like the quantum yield of the fluorophores. The FLAP ratio signal thus avoids a number of complexities and artifacts that plague interpretation of the signals provided by simpler techniques like FRAP [21].

It is sometimes convenient to express the FLAP signal in terms of the specific bleaching fractions of the G- and F-actin pools: 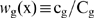, and 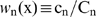. To do this we need only rewrite Eq. 8 in the form
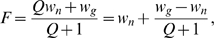
(9)where 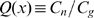 is the F-to-G ratio at a given cross section. For obvious reasons, the FLAP ratio close to the leading edge (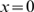) or inside the cap compartment (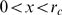) has a special significance and will be denoted by 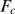. Generally, because the G
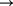
F reaction is greatly enhanced in the vicinity of the leading edge, one expects that 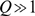 and 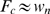. On the other hand, in the mid-lamella and the cell body most measurements indicate that the balance of actin polymerization usually tends to favor the G state. Thus, in such locations the usual situation is 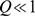 and 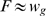.

## Results

We now examine a few numerical solutions of steady state and bleaching of our model lamella, starting with a benchmark case and then looking at departures from this as the model parameters are systematically varied. When interpreting these results, one should remember that perturbations in control parameters such as 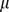 and 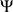 are simply indirect means of altering the phase velocities of the network and solvent.

### Case-0: A Benchmark Calculation

To keep matters as simple as possible for the benchmark case, we consider a situation where there is exact symmetry between the left and right halves of the computational domain, and where the basic input of energy is derived by specifying an enhanced rate of actin polymerization inside the two semi-circular “cap” compartments at either end of the domain. All other physical parameters of the cytoplasm (see next section) will be constant in space and time. The left-right symmetry of the benchmark case assures us that the forces on the computational domain are in global balance and that the velocity of the cell with respect to the substrate is zero. We therefore postpone the added complexity involved with balancing forces and calculating the gliding velocity of the model cell.

#### Case-0: Parameters

In addition to 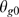 and the four geometric parameters that define our computational domain, our benchmark calculation involves exactly eight intensive physical parameters of the cytoplasm (Table 1). Values for three of these have been unambiguously determined by Zicha and coworkers. These are the equilibrium F/G actin ratio in the bulk cytoplasm 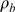, the G-actin lifetime 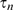, and the G-actin diffusion coefficient 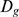.

Since our model conserves total actin volume, the value of 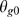 represents the average actin volume fraction not only at 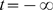 but also at every subsequent moment in time. Experimental values of the average actin content of T15 cells were not directly measured by Zicha et al., so we adopt instead the value of 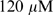 deduced from estimates by Abraham et al. [9] for similar cell lines. Using standard values for the density and molecular weight of globular actin 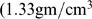, and 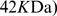, we can calculate that 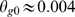, or 0.4% of cytoplasmic volume. Values reported in other types of amoeboid cells [22] indicate that the highest total actin concentration (1.2% of cytoplasmic volume) is found in neutrophils. Using this number in place of our current estimate has no substantial effect on any of our results.

In choosing the parameters governing the Darcy drag, the network viscosity and the network swelling behavior, our procedure is to use independent estimates previously published in other studies based on the RIF approach. The network-solvent drag (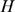) can be accurately estimated from known models of flows in fibro-porous media, given the viscosity of the aqueous phase of the cytoplasm and the diameter of actin filaments (see [12], [17] for details). For the specific network viscosity 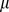, we use estimates obtained from micropipette aspiration of human neutrophils [17]. Finally, the network swelling energy 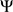 of 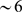 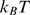 per monomer has also been determined in the neutrophil [17]. This represents a reasonable order of magnitude for the mechanical energy that can be stored per actin monomer at typical network densities, since it is on the order of the free energy released during the polymerization reaction. It is also equivalent to about 30% of the energy available from hydrolysis of one high energy phosphate bond of ATP. Naturally, if the swelling stress is much less than one 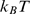 per monomer, than it is completely inconsequential, and if it becomes negative one would expect to observe coagulation, or bundling of the network phase.

What remains now is a single parameter governing the enhanced rate of actin polymerization in the two cap compartments. An extreme upper limit on this parameter is obtained by letting the value of 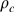 equal the ratio of the area of the cap compartment and the total domain area (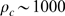). The resulting value (see Table 1) means that the rate of polymerization in the edge caps is increased by a factor of 10,000 over the value in the bulk cytoplasm. This level of enhancement means that in a static chemical equilibrium, approximately half the actin of the cell will become concentrated in the caps in the form of F-actin.

#### Case-0: Steady-state with Actin Treadmilling

**Figure 2:**
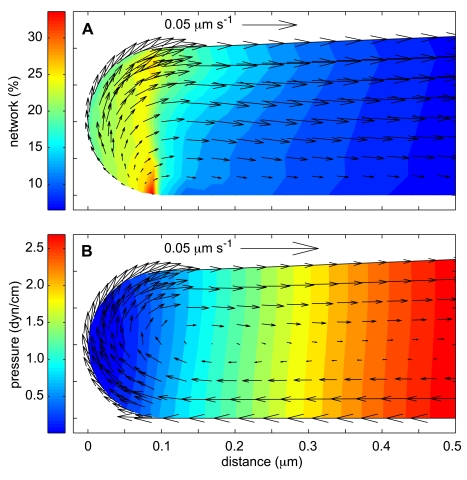
Steady state solutions at the lamella leading edge for Case-0 (benchmark). Parameter values are as indicated in Table 1. (A) Color contour plot of the network volume fraction with arrows indicating network velocity. The maximum volume fraction is at the ventral surface near the threshold of the cap compartment. The newly created network expands from the leading edge compartment, creating a retrograde flow of polymerized actin towards the main body of the cell. (B) Color contour of the pressure field with solvent velocity indicated by superimposed arrows. The cytosolic flow is entrained with the network along the dorsal surface, but is sucked forward by the low pressure of the cap for mass conservation.

To reach steady state we start with all actin in the G state and simulate the reaction and flow in the lamella for sufficient time so that the pressure and all densities and velocities become constant. The character of the steady state solution close to one of the cell edges is displayed in Fig. 2 (recall that there is left-right symmetry). The enhanced polymerization in the proximal cap compartment leads to a high network concentration which in turn causes swelling so that the network phase expands and flows towards the cell center (Fig. 2-A). The flow of polymerized material out of the caps leaves behind a void that causes a zone of low pressure to develop. The suction of this low-pressure zone, together with the Darcy drag exerted by the network, combine to create an eddy of cytosol that circulates dissolved material from the interior of the cell into the cap compartment along the lower boundary of the lamella and expels such material out of the cap along the upper surface (Fig. 2-B). As network flows towards the center, it leaves the region where the polymerization rate is enhanced. Consequently, the tendency to depolymerize is no longer counterbalanced by polymerization, and the network concentration decreases. This sets up a treadmilling cycle of polymerization at the edges, inward expansion, and depolymerization at the center which provides energy that drives a steady flow of network and cytosol that is stable.

**Figure 3:**
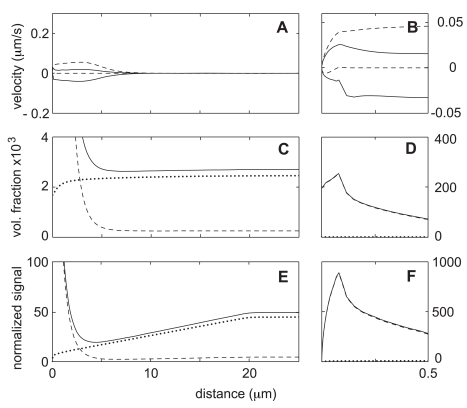
Steady state solutions for Case-0 (benchmark). Parameter values are as indicated in Table 1. Because of symmetry, results are shown only in the right half of the domain. Panels (A) and (B) show the network (*dashed line*) and the solvent (*solid line*) velocities at the top and bottom boundaries. Note that the flows are slow and extend only half the distance between the cap and the bleach zone (centered at 11.5 microns). Panels (C) and (D) show volume fractions of G-, F-, and total actin (*dotted line*, *dashed line*, and *solid line*). Panels (E) and (F) indicate predicted fluorescence intensity normalized to give 50% signal at the cell midpoint (*solid line*, see Fig. 4). Also shown is the breakdown of the intensity into its G- and F- components (*dotted line* and *dashed line*).

Fig. 3, -A and -B, show the character of the steady state flow for the base case of our model over a more extensive portion of the computational domain. Dashed lines show x-component of the network velocity, whereas solid lines show the x-component of the solvent velocity. Velocities which are positive or zero correspond to flows on the upper surface of the lamella, while negative velocities indicate flows at the lower boundary. Note that the network velocity is zero at the lower surface because the boundary conditions are “stick” at this surface. In contrast, the solvent phase has negligible viscosity and has a negative velocity at this surface (see Eq. 4).

Fig. 3-A indicates that the typical speeds of the network and solvent flow are in the range 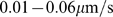. In the case of network flow, this is in excellent agreement with experimental values [23], [24]. On the other hand, simple estimates show that the computed flows are much slower than would be required to have a significant effect on G-actin transport. For example, if we assume a constant motion at the maximum velocity of 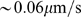, it would take 200 s for G-actin to move from the bleach zone to the leading edge. Even this is optimistic, since both the network and the solvent phase flows essentially stagnate and approach zero at a point several microns short of the bleach zone. Nevertheless, at least the basic concept of wide circulating channels of cytosolic flow through the actin gel of the leading lamella is a spontaneous and unforced prediction of even the benchmark version of our model, and one may certainly hope for flow fast enough to have some effect on transport of bleached G-actin for different parameters.

If we integrate over the whole cell, there is near equipartition of actin between the G and F pools for the steady state of our benchmark model. In the bulk of the cytoplasm, the volume fractions of the G- and F-actin equilibrate at 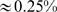 and 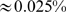, respectively (Fig. 3-C). This ratio, 10-to-1 in favor of the G state, is set by 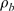 and was directly measured by Zicha et al. Since polymerization is increased inside the cap compartments, the situation there is reversed. The cap network volume fraction reaches a peak value of over 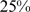, whereas the G-actin volume fraction is 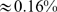 (Fig. 3-C, D). The horizontal location of the F-actin maximum is near the inner boundary of the cap, where the freshly delivered G-actin first enters the area of enhanced polymerization. The maximum is at the ventral surface of this boundary, where network expulsion from the compartment is impeded due to interaction with the substrate. Direct measurements of the average F-actin density at the leading edge are hard to find, but the average 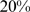 value we obtain from the present calculation is higher than a published theoretical estimate of 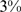 (40 mg/ml) [9]. This is not surprising since in this benchmark case we have deliberately chosen to set the cap polymerization activity at an upper limit.

Fig. 3 is a sketch of the typical experimental data for the total (YFP+CFP) fluorescence that summarizes the essence of what is presented in Zicha's Fig. 1 [1]. Following a path on the cell midline from the edge inward, the fluorescence signal increases rapidly to a maximum after a few microns. The intensity then remains high over a distance of about 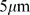 after which there is a sharp drop to a local minimum. After the minimum, the intensity slowly rises again along the length of the lamella until a second maximum is reached at the junction of the lamella and the cell body. This second maximum usually is located about 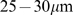 from the leading edge and has an amplitude about 60% of the peak value. At the middle of the cell body there is frequently an abrupt drop in intensity which is evidently an artifact caused by the presence of the nucleus.

Fig. 3-E and -F show the total fluorescence intensity in the benchmark model. To match the presentation of the experimental data, the volume fraction of G- or F-actin is integrated over the cell thickness and scaled so that the value of total actin at the cell midpoint corresponds to a 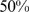 signal. The main features of the experimental profile are present: the sharp peak near the leading edge, followed by a minimum, and then a steady rise until the junction with the cell body. Since the model neglects the effect of the nucleus, the simulated intensity remains constant after this point. One may also note that the width of the predicted peak at the leading edge is too small and that the maximum intensity of this peak is too high. At least in part, these discrepancies are explained by the fact that the model assumes that the leading edge of the cell is a perfectly straight line, whereas in reality the leading edge typically meanders back and forth by 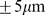 around a mean position. Since the experimental data are obtained by averaging an 8 micron-wide strip across the lamella, the meanders will inevitably cause some blurring or smearing out of the fluorescence signal. Thus the broad multi-component distribution of the experimental signal could be thought of as a convolution or weighed sum of numerous sharper peaks each of which is individually similar in shape to the one obtained in our simulation.

#### Case-0: A Weak FLAP Signal

The steady state solution for Case-0 (see previous section) will now be used as the backdrop for a numerical FLAP experiment. At the initial time, a zone extending between 10 to 13 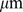 from the left edge of the lamella is “bleached” by instantly converting all actin (both F and G) from the unbleached to the bleached states. The bleaching process is then continued for duration 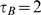 s. At the end of the bleaching phase, the total bleached actin in the cell consists of all actin monomers that were initially in the bleach zone together with any actin monomers that entered the bleaching zone as a result of diffusion or convection processes during some stage of the 2 s interval. Integrating the total amount of bleached actin over the whole cell at the end of bleaching we find that approximately 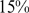 of actin monomers have been labeled, exactly in accord with the results of Zicha et al. Bleached molecules are created only during the bleaching, and the total mass of such molecules remains fixed at subsequent times. Thus at very long times, when bleached and non-bleached monomers are well mixed, the FLAP ratio will approach a uniform value of about 0.15 throughout the cell.

**Figure 4:**
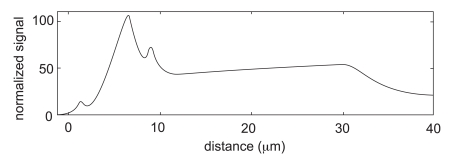
Sketch of the typical actin fluorescence. The fluorescence intensity is shown as a function of distance from the leading edge in a T15 cell (adapted from [1]). The fluorescence is averaged over a strip about 8 microns wide parallel to the cell long axis and normalized so that value at the base of the lamella is 50%.

Fig. 4-C through -F show the detailed distribution of the bleached monomer F and G fractions at the end of the bleaching process (solid line). Also shown is the computed FLAP signal at the end of bleaching (Fig. 4-A and -B). Note that inside the bleaching zone the FLAP signal is necessarily equal to 1 at 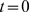 since all yellow fluorophores are bleached in this region. Note also that G-actin can diffuse a typical distance 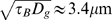 over interval 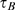 whereas F-actin, which moves only via convection, can move no more than 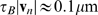. This explains why, even at 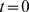, the bleached G-actin has already spread far outside the bleaching zone whereas the F-actin remains essentially fixed.

The fact that the bleaching phase has a finite duration and that transport toward the leading edge begins at the start of the bleaching phase is important for interpreting the observed time intervals for transport between the bleaching zone and the leading edge in the experiments of Zicha et al. The reported two second time interval for development of the leading edge signal can be somewhat misleading because this corresponds only to the time from the end of the bleaching. If we consider that most bleached monomers are created at or near the start of bleaching and that such monomers have four seconds to reach the front, then the reported result is less startling.

Fig. 4-C through -F also show the distribution of labeled F- and G-actin 2, 5, and 15 s after bleaching ends. It is apparent that at the 2 s point, the distribution of G-actin is bell-shaped with standard deviation of about 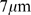. The tails of this distribution are sufficient for a considerable amount of bleached G-actin to reach the closer of the two edge compartments. Since G-actin at this edge is converted very rapidly into F-actin, the result is a very sharp local maximum of the bleached F-actin inside the front cap. Nevertheless, the mass of the bleached material in the cap is very small, and most of the bleached F-actin is still located close to the bleaching strip where it was initially created.

The FLAP ratio signal 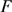 at 2, 5, and 15 s is shown in Fig. 4, -A and -B. The maximum 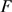 is still in the bleaching zone. However, because of depolymerization of F-Actin and diffusion of unbleached monomer, the signal here is no longer 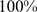 (the maximum is about 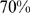 at 2 seconds and 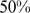 at 5 seconds). This decay in the bleaching zone is in accord with the results of Zicha *et al.*, and formed the basis for the estimates of F-actin lifetime and diffusion constant used in our simulations. The more interesting aspect of our calculated FLAP signals is the existence of a very weak local maximum at the lamella edge at both 2 and 5 s after bleaching. These are best seen at the expanded scale of Fig. 3-B. The magnitude of the leading edge maximum is 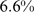 at 2 s and 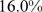 at 5 s and reaches a maximum of 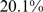 at 15 s. After this it decreases very gradually to the final value of 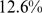.

As a summary, we may conclude that the FLAP signal predicted by Case-0 resembles, at least qualitatively, the results reported for the “pure diffusion” calculations of Zicha et al. Case-0 can produce some small suggestion of special dynamics at the leading edge but it cannot explain the reported occurrence of 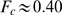 at the 2 s time point, or indeed at any time. Thus we can confirm that at least the quantitative results obtained by Zicha and coworkers are not the sort of thing one expects to see as a routine matter. On the other hand, the qualitative properties of Case-0 suggest that it may be possible to obtain results closer to what is seen in experiment by simply adjusting the solvent and network flow fields. This is what we will attempt to do in the next section.

### Case-1: A Model Cell with Lower Cytoskeletal Viscosity

As was pointed out by Zicha and coworkers, sluggish diffusive transport of globular actin from the bleaching zone to the leading edge affords a simple and direct explanation for the failure of Case-0. Since the distances to be covered and the diffusion constant of G-Actin are known quantities not amenable to much adjustment, an improved model in this regard would necessarily rely on some change in the rate of convective transport. Such transport is in fact happening in Case-0, but as we have discussed, it is very slow. In terms of our quasi-dynamical model, the simplest way to speed things up is to reduce the network viscosity while leaving all other parameters of the model unchanged. Indeed, since the coefficient of network viscosity listed in Table 1 is derived from studies of human neutrophils, the idea of a somewhat different viscosity in the case of T15 cells is reasonable. Therefore, for Case-1 of our model we will consider the consequences of reducing the base viscosity by a factor of ten (Table 2).

**Table 2:** Definition of cases.

Case 0	Benchmark, see Table 1
Case 1	Reduced viscosity, 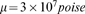
Case 2 (Appendix S1)	Increased cap swelling, 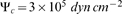
Case 3 (Appendix S1)	Gliding cell, 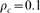 in the right cap

#### Case-1: A Steady-state with Rapid Retrograde Flow of Cytoskeleton

**Figure 5:**
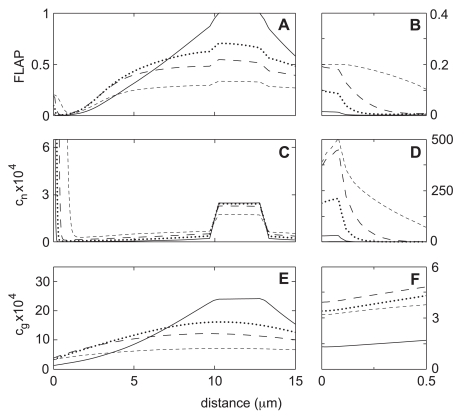
FLAP calculation for Case-0 (benchmark). Panels (A) and (B): Spatial distribution of computed FLAP intensity immediately after the 2-second bleaching period (*solid line*) and at 2, 5, and 15 s (*dotted* line, *long-dash* line, and *short-dash* line). No significant FLAP is seen at the leading edge at 2 s, and the maximum value never exceeds 0.2, similar to the result obtained with the diffusion-only model in Zicha at al. Panels (C) and (D): The thickness average of the bleached F-actin (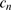). Notice that despite the absence of a strong leading edge FLAP signal at 2 s, there is a significant amount of labeled F-actin present. Panels (E) and (F): The thickness average of the bleached G-actin (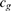).

After making this change and allowing sufficient time for equilibration, Case-1 yields the flows and mass distributions that are summarized in Fig. 5. As indicated by panels -A and -B, there is a five-fold increase in the peak speed of both the network retrograde flow and the solvent recirculation flow. In addition, the size of the zone covered by the solvent circulation is greatly increased, and is now more than sufficient to convey matter for the whole distance between the bleaching zone and the leading edge at near maximum speeds. Thus the desired kinematic result of reducing the network viscosity has been achieved.

Of course one should not fail to notice that the network flow predicted for Case-1 (i.e.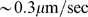) is now so fast that it exceeds what is observed for most motile cells by an order of magnitude [23], [24]. On the other hand, this prodigious flow rate is not completely beyond reason since there has been at least one well-documented study where such rapid retrograde flow was observed [25]. We should also notice that while fast compared to experimental measurements, the speed of 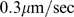 is much slower than that proposed by Zicha et al. for their calculations testing the advection hypothesis. This velocity also clearly falls short of what is needed to move material from the bleach zone to the edge in just two seconds. Thus, at best, Case-1 only provides improved advective “boosting” to what is still diffusion-dominated transport.

The viscosity decrease in Case-1 has other significant effects on the steady state solution of our model. For example, since network exits the leading edge more efficiently, the peak of the F-actin density at the leading edge is much lower and wider than in Case-0 (compare Fig. 3-D and Fig. 5-D). The enhanced F-actin transport away from the edge is even enough to cause a small increase in the amount of F-actin in the bleaching zone (compare panels -C in Fig. 3 and Fig. 5). Feedback from these significant changes in F-actin distribution then causes perturbations in the G-actin distribution. First, since F-actin produced in the edge moves further before spontaneous conversion back to the G-form, there is a higher steady state level of G-actin in the cell body and in the bleach zone (see also Table 3). Moreover, on average, the G-actin released from F has a longer distance to travel in order to cycle back to the cap compartment, so the steady state G-actin at the leading edge is lower than seen in Case-0. The increased G-actin in the bleach zone is important when it comes to a FLAP experiment because it allows for bigger production of bleached monomers. The decrease in G-actin and F-actin at the leading edge is also important since the FLAP signal is inversely related to these quantities (see Eq. 9).

**Table 3:** Comparison of cases.

Value		C-0	C-1	C-2 (Appendix S1)	C-3 (Appendix S1)	
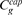	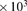	1.71	1.20	1.63	1.32	a
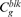	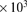	2.41	2.80	2.46	3.06	b
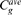	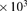	2.41	2.77	2.46	3.15	c
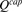		131	32.7	25.0	43.4	d
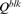	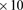	1.02	1.93	1.02	1.65	e
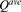	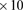	6.62	4.41	6.25	2.65	f
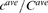	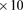	1.26	1.52	1.29	1.64	g
FLAP (0)	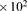	0.8	5.1	4.2	4.2	
FLAP (2)	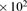	6.6	25.9	18.9	23.8	
FLAP (5)	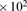	16.0	37.8	24.9	37.5	
FLAP (15)	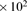	20.1	30.1	20.1	30.4	
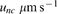	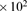	2.4	11.0	12.0	8.1	h

^*a*^height average of 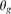 calculated at 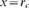.

^*b*^height average of 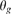 calculated at 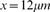.

^*c*^volume integral of 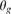 over the cell, divided by cell volume, 33.90 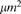.

^*d*^ 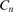/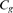 at 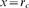.

^*e*^ 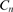/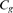 at 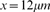.

^*f*^ 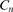/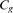 averaged over the entire cell.

^*g*^average fraction of bleached actin molecules in the total actin pool.

^*h*^flux average of the network velocity at 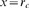.

#### Case-1: A Stronger FLAP Signal than the Benchmark Case-0

**Figure 6:**
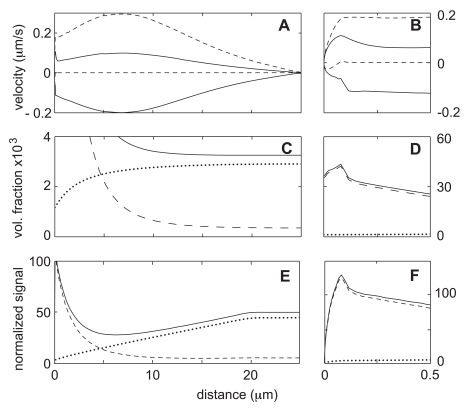
Steady state solutions for Case-1 (lower network viscosity). Parameter values are as in the benchmark case (see Table 1), except that the viscosity is lowered by a factor of 10. Panels (A) and (B) show the network (*dashed line*) and the solvent (*solid line*) velocities at the top and bottom boundaries. As a result of lower network viscosity, the flows are much faster, and the area of strong flow now extends as far as the junction of the lamella with the central plateau region. Panels (C) and (D) show volume fractions of G-, F-, and total actin (*dotted line*, *dashed line*, and *solid line*). Panels (E) and (F) indicate predicted fluorescence intensity normalized to give 50% signal at the cell midpoint (*solid line*). Also shown is the breakdown of the intensity into its G- and F- components (*dotted line* and *dashed line*). Notice that the peak intensity at the leading edge is decreased relative to the benchmark case whereas the width of the peak is increased. This results in an intensity profile that is in a better agreement with the experimental results reported by Zicha et al. (see Fig 4).

Fig. 6 shows the new FLAP signals calculated after reduction of the network viscosity in a way matched with the earlier calculation of FLAP in Case-0 (Fig. 4). We find that already at 2 s, about 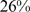 of the actin at the leading edge has somehow been transported from the bleach zone (dotted line in panel-B). At 5 s post-bleach, the leading edge signal increases to 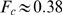, but thereafter it begins to decrease, and is down to about 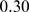 15 s after bleaching. While the 2 s signal in this calculation is impressive, it is still short of the 40% signals at two seconds reported by Zicha et al. Nevertheless, the trend is clear, and it is easy enough to match the experimental value exactly by further adjustments of the viscosity (data not shown).

**Figure 7:**
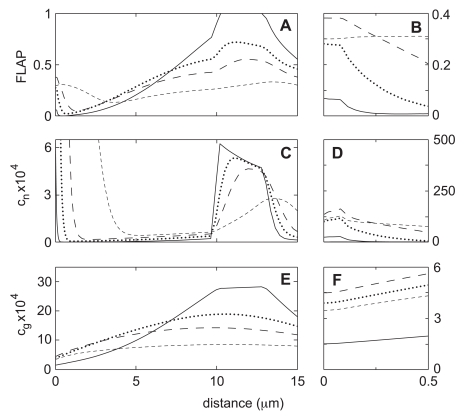
FLAP calculation for Case-1 (lower network viscosity). Panels (A) and (B) indicate spatial distribution of the FLAP signal immediately after the 2 s bleaching period (*solid line*) as well at 2, 5, and 15 s (*dotted line*, *long-dash line*, and *short-dash line*). The signal at the leading edge is as high as 0.25 at 2 s, and increases to 0.38 at 5 s. At 15 s, the FLAP has begun to equilibrate towards a uniform final distribution. Panels (C) and (D) show the thickness average of the bleached F-actin volume fraction (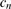). The advection of F-actin is sufficient to produce observable transport of the F-actin out of the bleaching zone. Note that the amount of bleached F-actin at the leading edge is smaller than in Case-0, yet the FLAP signal is much higher. This illustrates the importance of the background actin density, since the FLAP signal is measured relative to this level. Panels (E) and (F) show the distribution of the bleached G-actin (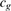). Note that despite the substantial increase in flow rates and the changes in the distribution of F-actin, the distribution of bleached G-actin is not significantly changed compared to the benchmark case. This is not what one would expect if the improved FLAP signal is due to changes in G-actin transport.

With regard to Fig. 6, one may also note some additional subtle differences in the FLAP dynamics between Case-0 and Case-1. For example, the distribution and temporal dynamics of the F-actin in the bleaching zone (panel -C) are quite distinct in the two cases. In particular, at later times (5 and 15 s), the peak of bleached F-actin in Case-1 has translated and dispersed toward the rear of the cell while there is no visible motion in Case-0. These changes can be traced to the fact that the retrograde flow in Case-1 has a bigger range and extends well past the bleach zone.

#### Case-1: Is the Stronger FLAP Signal a Result of Increased Convection?

At this point one can be excused for thinking that the properties of Case-1 confirm the predictions of the advection hypothesis. The results suggest that decreasing the network viscosity has increased the speed and range of the cytosolic circulating flow driving the emergence of a strong leading edge FLAP signal at 2 s after bleaching. However, some suspicion that something is wrong with this interpretation arises from the fact that there is almost no difference in the distribution of bleached G-actin in Case-0 and Case-1 (compare panel-E or panel-F in Figs. 4 and 6). Therefore, before accepting the advection hypothesis, it is still necessary to consider controls demonstrating that the improved FLAP signal in Case-1 is not the consequence of some other unintended side effect of changing the network viscosity. To provide such control, we have repeated the Case-1 calculation after numerically eliminating the advective term in the transport equation for G-actin. If the advection hypothesis is correct, then obviously this calculation should give results similar to those obtained in Case-0 (i.e. a big decrease in the 2 s FLAP signal at the tip). Unexpectedly however, numerical elimination of G-actin advection causes almost no difference in the behavior of Case-1. Indeed, the FLAP values at the leading edge for Case-1 with and without boosting of G-actin advection are equally enhanced relative to what is found in Case-0 and differ from each other by not more than 1.5% at all time points (data not shown).

We must therefore conclude that Case-1 does predict something close to the data of Zicha et al., but that the reason this model works is unrelated to the original idea motivating its fomulation. Control calculations such as the one just described demonstrate conclusively that advective transport of G-actin by the cytosolic flow is completely unimportant in Case-1 and that, just as with Case-0, transport is largely dominated by simple diffusion. This conclusion is valid not only for these cases, but holds even for cases with much lower viscosity where the speed of solvent circulating flow is increased by an additional factor of ten. In fact, we find that cytosolic flow seems to be negligible for FLAP signals in all physically reasonable sectors of parameter space.

The deeper physical explanation for the failure of the advection hypothesis can be appreciated if we consider the typical motion of a single bleached G-actin molecule that is created at 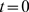, and then immediately picked up from the bleached region by the forward cytosolic flow at the ventral cell surface. According to the advection hypothesis, this molecule diffuses back and forth but essentially remains in the forward-directed channel long enough to make considerable progress toward the leading edge. This would be a valid picture if the forward-directed solvent channel were enclosed by impermeable walls, or if the stream were very wide, or if there were some other factor(s) to prevent the diffusing molecule from escaping the forward-bound flow. However in reality, there are no well-defined walls in an actin gel, and molecules of G-actin can therefore freely diffuse through the gel pores in the vertical direction as well as horizontally. This means that after a certain period of forward advection, a typical G-actin molecule will leave the forward stream and enter the backward-flowing stream. It will then advect in the centripetal direction for a short time before once again entering the forward stream and so forth. The characteristic time of the molecular diffusion across the thickness of the lamella is 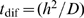, and has an upper bound of about 0.1 seconds (Table 1). In contrast, the time scale for advective transport is 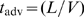, where 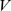 is a characteristic velocity for the cytosolic flow, and 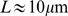 is the distance from the leading edge to the bleaching zone. Unless the advection velocity is 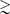
10 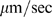, advection is much slower than vertical diffusive mixing. This means that, for practical flow speeds, a given monomer jumps between streams many times so that on a macroscopic scale, the net advective contribution to the overall transport towards the leading edge averages to the net forward flow of solvent which is very close to zero. This schema is a well know feature of many systems where transport from competing microscopic flows is averaged by transverse diffusion (see [26] for a full quantitative discussion of Taylor dispersion).

### The Dilatation Hypothesis

The analysis of the previous sections demonstrates that transport of G-actin in both Case-0 and Case-1 of our model is dominated by simple diffusion and that transport via cytosolic flow is negligible. Thus advective transport cannot be the reason for the increased leading edge FLAP signal in Case-1 vs Case-0. In fact, it seems clear that the enhanced FLAP signal produced by reduced viscosity has nothing to do with transport of G-actin and is actually due to a local effect on the dynamics of actin in the cap compartment itself. A clue to the nature of this local change comes from the fact that reduced network viscosity greatly increases retrograde flow of F-actin. Since the boundary conditions demand that F-actin flow vanishes at the leading edge, this means that the F-actin in the cap is expanding or dilating at a greater rate when we reduce viscosity. This rate of dilation is potentially important because it shortens the turnover timescale for newly created F-actin filaments. We may therefore suggest as a hypothetical rule that the leading edge FLAP ratio is a strongly increasing function of the local mechanical dilatation rate of the F-actin in the cap compartment. For lack of a better term, we will call this proposal the “dilatation hypothesis”.

A measure of the overall network dilatation in the cap is obtained by averaging the point-wise divergence of the network velocity. Using the divergence theorem, this means that the leading edge dilatation can be defined in terms of either a volume integral or a surface integral:
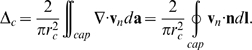
(10)Moreover, since 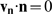 on the exterior surfaces of the cap, the outflow integral becomes 
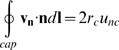
, where 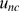 is the average of the x-component of 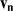 over a vertical cross-section at 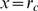. Further substitution then yields 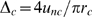. This last fomula can be used to calculate 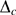 for various cases of interest using tabulated values of 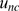 (Table 3).

If we hold geometry fixed, the dilatation hypothesis implies that the FLAP signal at the leading edge is enhanced or reduced directly in response to changes in the state of motion of the network. Equivalently, one might say that a perturbation of 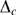 controls the FLAP signal in the same fashion regardless of how one creates the perturbation. This is a necessary property of any reasonable hypothesis because, as we have indicated before, FLAP signals are ultimately governed solely by the distribution of labeled and unlabeled actin monomers, and the deeper properties of the cytoskeleton (e.g. three control coefficients, 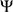, 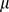, and 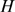) can only influence these signals indirectly, by determining phase velocities or reaction rates.

In Appendix S1, we present a detailed discussion of two additional numerical experiments (Cases -2 and -3) designed to empirically test the validity of the dilatation hypothesis (the key properties of these cases are summarized in Table 2 and Table 3). In Case-2, we examine the effects of a perturbation in the network swelling stress that mimics what would happen if myosin II contractility is decreased at the cell edges relative to the bulk cytoplasm. The result is a very localized increase of the retrograde flow speed inside the edge caps which increases 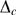 even while the network and solvent flow in the bulk of the computational domain remains very similar to that of the benchmark case. The FLAP signals after this perturbation are greatly increased supporting the dilatation hypothesis and providing an explanation for why the leading edge FLAP signal is sensitive to inhibitors of myosin II. In Case-3, we consider a perturbation that breaks the left-right symmetry of the benchmark model and causes the entire computational domain to begin translational motion that eventually settles into a steady state with constant velocity. Once again, the simulation of FLAP experiments under these circumstances confirms the predictions of the dilatation hypothesis and provides an explanation for why FLAP signals are enhanced on advancing cell margins.

## Discussion

We have here been concerned with understanding the FLAP experiments of Zicha et al. in which labeled G-actin generated photochemically 10–15 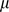
m from the front of the cell appears at segments of the leading edge after a delay of only 2 to 4 seconds. At peak, the amount of labeled monomer in these rims constituted 20–40% of the total actin present. The segments of the edge showing high FLAP activity were mainly associated with zones of active protrusion, and in addition the activity was sensitive to inhibitors of myosin II.

Zicha et al. argued that the observed transport of labeled monomer from the bleach zone to the rims is too fast to be explained by simple diffusion and they inferred the need for a specialized advective transport mechanism. They further indicated that this mechanism probably involved circulation or flow of cytosol through small channels in the actin matrix connecting the bleach zone and advancing edges of the lamella. The implication of this “advection hypothesis” is that transport and supply of actin monomer in these channels is an important regulatory influence on the motion of the cell margin.

The advection hypothesis is certainly a logical possibility and our analysis has indicated that channels of rapidly flowing cytosol actually do spontaneously form in the actin gel, and that these circulate material through the leading edge compartment much in the manner suggested (see Fig. 2). On the other hand, we also find that the flows we observe do not really work as a significant modality of G-actin transport because the walls of channels inside a porous gel are highly permeable and present a negligible barrier to diffusion. As a result, mixing of material inside and outside the channels is sufficiently rapid to negate the possibility of effective advective transport along the channel length. This conclusion is quite robust and remains valid even for unrealistic assumptions that greatly favor advective transport, e.g. even if the channels are very wide (e.g. half the thickness of the lamella) and even if the flow in such wide channels is exceedingly fast (e.g. 10 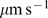).

Our results also demonstrate that for the typical distances and time scales governing the cytoskeleton of a T15 cell, simple diffusion is actually quite sufficient to explain the rapid emergence of a strong FLAP signal at the leading edge. As far as the correlation of early FLAP and protrusion, the simplest explanation is based completely on the effects of dilatation rate. This is what we have called the “dilatation hypothesis” and it works as follows: 1) The FLAP signal in the cap compartment is masked if the compartment is clogged by a high density of F-actin. 2) The masking is particularly effective if the filaments are stationary and turning over in a sluggish fashion so that they are difficult to label. 3) The cap dilatation rate decreases the F/G ratio of the cap and thus relaxes this preexisting FLAP inhibition. 4) Protrusion increases dilatation, which means the FLAP signal is elevated on protruding segments.

Given our analysis, the FLAP signal is highly sensitive to several parameters that characterize cytoskeletal dynamics in an exceedingly small compartment at the leading edge where crutial events are believed to happen. Moreover, although the temporal resolution of FLAP is currently quite poor, this should be easily improved by application of faster and more sensitive scanning technologies. The uniquely non-invasive character of FLAP measurements may in principle alow for studies of living cells without effecting their ongoing behavior in even the slightest way. Finally, there is no reason why the method should be is restricted to actin dynamics. In theory, any protein of the cytoskeleton could be studied in a similar way. Hopefully, the current work will encourage additional experiments and applications of this promissing technique.

## Supporting Information

Appendix S1 Additional numerical experiments.
(0.52 MB PDF)
