# Supplementary material for: Analysis of Actin FLAP Dynamics in the Leading Lamella
Source: PLoS One. 2010 Apr 15;5(4):e10082. doi: 10.1371/journal.pone.0010082 (PMC2855347; doi:10.1371/journal.pone.0010082)
Supplement: Appendix S1 — Additional numerical experiments. (0.52 MB PDF) [file pone.0010082.s001.pdf]

Appendix S1

Case-2: Perturbation of the Swelling Stress

We will now consider a further numerical experiment in which the predictions of the dilatation hypothesis and the advection hypotheses are contradictory. To achieve this, we propose to locally increase the network swelling energy inside the left and right cap compartments by a factor of 10, while keeping Ψ outside the caps and all other parameters fixed at the benchmark levels.

The panels of Fig. A1 show the steady state network and solvent velocities, as well as the various volume fractions for Case-2. These panels are directly comparable to the corresponding panels in Figs. 3 and 6 which show similar plots for Case-0 and Case-1, respectively. As one might expect, the localized nature of the swelling perturbation vs benchmark induces changes in the flows and mass distributions that are also localized. For example, comparison of panel-A in Fig. 3 and Fig. A1 shows that the network and solvent velocities for the present case are very close to the benchmark values at locations outside of the cap compartment. In contrast, comparison of the results in panel-B in these figures shows that the increased swelling energy inside the caps has induced big changes in the cap dilatation. More precisely, the value of u_{nc} in the present case is 5 times higher than in the benchmark. Thus the advection hypothesis predicts similar leading edge FLAP signals for benchmark case and Case-2, whereas the dilatation conjecture predicts a much stronger signal for Case-2 vs benchmark.

In Fig. A2 we show the FLAP calculation for Case-2, which provides the critical test. We observe that in Case-2 the leading edge FLAP at 2 s post-bleach is about 19%, rises to 25% at 5 s, and then falls back to 20% at 15 s. The reader may confirm that these properties, as well as the shape of the FLAP signals in Case-2, match well with Case-1 and not at all with Case-0. Thus the test is conclusively in favor of the dilatation hypothesis.

As an aside, one might add here that the rapid expulsion of actin from the edge compartment in Case-2 means that the F-actin concentration is at a maximum not at the edge but only at some distance behind the threshold. This precise behavior has in fact been directly observed experimentally by Soo and Theriot [1] in the tail of *Listeria monocytogenes*. The averaged actin tail density plots for *Listeria monocytogenes* consistently show a global maximum at a distance on the order of $1\,\mu$m from the rear end of the bacteria. This feature in common with our simulations suggests that the mechano-chemical interaction between the filaments and the cell wall of listeria or a membrane of the leading edge results in active network expansion, and that the intensity of this expansion rapidly reduces with distance from the cell wall.

We should also pause to consider the fact that the effective value of Ψ reflects a summation of microscopic repulsive and contractile interactions (see discussion of momentum conservation). Furthermore, retrograde flow and cap dilatation respond to gradients of the product $\Psi\theta_n$, rather than to absolute values of either factor. In other words, if $\Psi > 0$, then increased retrograde flow can be induced by increasing Ψ or θ_n at the front of the cell, or by decreasing them at the back, or by a combination of both. Accordingly, simulations with lower swelling energy in the bulk lamella increases dilatation and FLAP as expected (data not shown). This insight is important because myosin II tends to be excluded from the leading edge and is mainly distributed in the cell body and tail [2]. As a result, inhibitors of myosin II are expected to increase the effective value of Ψ in the bulk cytoplasm and thereby decrease the retrograde flow. Thus the dilatation hypothesis provides a ready explanation of why inhibitors of myosin II decrease leading edge FLAP signals. If this interpretation is correct, it serves as evidence that retrograde flow is driven by a combination of effects: partly by swelling forces or polymerization forces pushing at the front, and partly by contractions at the base of the lamella which pull the cytoskeleton backward [3, 4]. Biological systems frequently make use of such redundant layers of design.

Case-3: The Case of a Gliding Cell

For T15 cells, as with other fibroblastic cells [5], the leading edge at any given time can be divided into segments that are advancing and other segments that are retracting. These segments gradually change from one class to another like successive waves lapping a beach; overall progress of the cell occurs because, like an incoming tide, the advancing segments have a slight statistical advantage. Based on observation of many individual cells, Zicha et al. found that two seconds after bleaching, advancing portions of the leading edge were prone to show high FLAP, while retracting segments showed weak signals. An example of this effect is clearly seen by comparing the 2 s FLAP signal image with a protrusion-retraction diagram of the same cell (e.g. Fig 3-C and -D of Zicha et al.). To account for this FLAP/protrusion correlation in terms of their advection hypothesis, Zicha et al. proposed that protrusion of an edge segment was conditional on a high local rate of F-actin assembly and therefore also on the rapid transport of G-actin to the protruding margin.

Since the correlation of high FLAP signals with protrusive activity is clearly a crucial bit of evidence, we now consider a situation (Case-3) designed to extend our prior conclusions so as to encompass circumstances of a moving cell. In particular, we will be concerned to see if the advective transport of G-actin towards a portion of the cell margin is enhanced or facilitated by protrusion. We will also consider whether diffusion is really insufficient to supply enough actin to support observed rates of margin protrusion. Further, we will ask if the dilatation hypothesis can explain the FLAP/protrusion correlation.

Case-3: Asymmetry Drives Migration

Before presenting the details of the Case-3 calculation, we briefly review important mechanical principles that determine cell motion in our model. To crawl, a cell (and in particular a T15 cell) must exert propulsive traction forces that thrust backward on the underlying substrate. When an adherent fibroblastic cell becomes polarized and initiates directional motion, these propulsive traction forces can be observed in a thin zone about 5 microns wide at the leading edge. Hence the leading edge acts as a locomotive that "pulls" the body of the cell forward [6]. At the same time, the trailing edge of the cell drags against the substrate producing a frictional load that resists the forward motion. Since the inertia of a cell is extremely small (low Reynolds number limit, see [7]), the sum of propulsive forces at the cell front is equal and opposite to the sum of the passive drag forces acting at the back.

Examination of the detailed network flow and density distribution of Case-0 (Fig. 2-A) illustrates how the traction forces on the substrate are related to movements of the cytoskeletal network. Near the ventral surface, the network velocity approaches zero, but the velocity gradient in a vertical direction has a non-zero value. This means that a significant viscous shear stress is acting on the lower boundary. The value of this stress, $\mu\theta_n \partial v_n/\partial y$, is one and the same as the active cellular traction stress being exerted on the substrate. For Case-0, the traction forces near each of the two caps thrust the underlying substrate inward, towards the cell center. Thus, by action and reaction, the edges of the cell are stretched in opposite directions which induces a static tension in the cell body.

In order to convert Case-0 into a case where the cell will move, it is necessary and sufficient to break the left-right symmetry in some fashion. For Case-3 of our model, we achieve this by simply "deactivating" one of the cell edges so that the rate of actin assembly in the associated cap compartment is the same as in the bulk cytoplasm. The velocity fields and the associated viscous shear stresses on the substrate become asymmetric. The cell computational domain is pulled forward by the remaining "active" cap, whereas the deactivated cap drags against the substrate. Since the domain shape is constant, the motion must be a uniform translation not unlike that of a keratocyte, and the velocity of this motion will be the *gliding velocity*, U. The actual value of U is not explicitly known, but is implicitly defined by the governing

equations and the requirement that the integral of the active and frictional forces on the substrate must be precisely zero at each instant of time.

Case-3: Protrusion Enhances Dilatation

Although the details of the numerical methodology are somewhat complex, running the simulations of Case-3 is essentially a matter of starting with arbitrary initial data and continuing until all the model variables, including the gliding velocity, the G- and F-actin distributions, and the associated velocity and pressure fields reach a steady state. For a given set of parameters such a steady state is unique and is reached in approximately 2 minutes if the initial conditions correspond to the steady state of Case-0. The final steady state value of the cell velocity in Case-3 is $-0.14\,\mu$m/s. This value is much bigger than the time-averaged migration velocity of T15 cells observed by Zicha *et al.* (about 4.4 nm/s). On the other hand, this value of U is not very different from that seen in very rapidly moving cells like the fish keratocyte [3] and the neutrophil [8]. In addition, the $0.14\,\mu$m/s value is close to transient velocities observed for the outward surge of a single cycle of lamella protrusion in T15 cells (see Fig. 3 in Zicha at al.). It is probably best to loosely interpret Case-3 as representing the dynamics only within a transverse section of a cell taken perpendicular to an advancing portion of the leading edge during the high point of a surge of outward motion.

Fig. A3 and Fig. A4 show details of the steady state solution of Case-3 near the leading and the trailing edges of the cell. These can be directly compared to similar figures for Case-0. All the velocities in these figures are in the frame of reference of the moving cell. Since the gliding velocity is negative, the network velocity (Panels -A, arrows) at the ventral surface of the computational domain is $+0.14\,\mu$m/s. The forward motion of the cell leads to the polymerized actin leaving the leading edge cap at a faster rate than in Case-0 so that the dilatation rate of the F-actin in the cap compartment is elevated. Unlike the leading edge cap, the trailing edge is governed by polymerization rates and swelling pressure identical to those in the main cell body. Therefore, the filament flow here is mainly a result of the cell motion over the substrate; the friction at the lower boundary drags the polymer backwards until it is trapped and compressed into a plug at the end cap. Swirling flows of cytosol are apparent at the leading and trailing edge (Fig. A3 and Fig. A4, Panels -B, arrows). At the leading edge, cytosol enters the top of the cap with maximum velocity of $-0.015\,\mu$m/s and exits along the ventral surface at velocity $0.08\,\mu$m/s. Other factors being constant, the cytosolic circulation within the leading edge is about 4 times faster in the case of a protruding edge (Case-3) as compared with a stationary edge (Case-0). The flow also has the opposite sense, i.e. counterclockwise instead of clockwise.

An important feature of the flow is that in the frame of the gliding cell, protrusion does not lead to any net outward or inward transport of cytosolic volume. Of course, such transport is obvious and necessary in the laboratory frame, since the motion requires that cytoplasmic volume to be added at the leading edge and removed from the tail. However, in the moving frame the net forward streaming is canceled by the motion of the observer and what remains is a circulation flow with equal inward and outward arms entering and leaving the cap compartment. This means that, just as in a stationary cell, the process of Taylor dispersion impedes advection. We find that the main (but small) effect of edge protrusion on G-actin transport is actually negative: the bleaching zone is stationary with respect to the substrate and therefore the outward motion of the edge gradually increases the separation with the site of final actin assembly, creating a larger hurdle to deliver the labeled material to the leading edge.

Fig. A5 shows more extensive plots of velocity and volume fractions for the steady state solution of Case-3. As before, all the velocities are in the frame of the gliding cell, so the network steady state velocity at the ventral surface is constant (Fig. A5, -A, -D, and -E). At the dorsal surface, the retrograde flow is faster, reaching $0.18\,\mu$m/s about $9\,\mu$m behind the leading edge, which means that in the laboratory

frame of reference the maximum retrograde flow velocity is 0.04 μm/s, close to that in Case-0 and Case-2. Fig. A5, -B, -F, and -G, show the detailed averaged volume fraction profiles, while Fig. A5-C displays the predicted fluorescence profiles. The volume fraction and the fluorescence profiles are similar to those obtained for Case-1 at the leading edge.

Case-3: Protrusion Enhances the FLAP Signal

Since Case-3 leads to significant dilatation of F-actin near the front cap, we expect a rapid increase in the leading edge FLAP signal. Simulations confirm this and the FLAP signal for Case-3 (Fig. A6) looks similar to that of the Case-1 (Fig. 7), with 23% FLAP ratio signal at 2 s, and 37% at 5 s.

We are now in position to interpret the observed correlation between zones of edge protrusion and higher FLAP signal. We have found that by starting from the stalled cell of Case-0 and simply "letting go" of the trailing edge, the FLAP signal in the leading edge is dramatically increased. For one thing, this shows that a simple change in the local conditions at the rear of the cell is sufficient to dramatically and rapidly change the FLAP signal at the leading edge. This happens simply because the change at the tail eventually increases protrusion and causes enhanced dilatation or stretching of the actin in the leading cap compartment. A similar enhancement of FLAP would be predicted after a tail detachment or if adhesion at the tail were suddenly decreased. Regardless of any other factors at play, increased rates of protrusion will by themselves enhance the leading edge FLAP signal simply by increasing the dilatation rate of the network. Thus the dilatation hypothesis explains the FLAP/protrusion correlation because it predicts that FLAP acts as a trailing indicator of protrusion. In other words, the correlation works because protrusion directly in and of itself enhances the FLAP signal and not because increased actin assembly, or some other third process enhances both.

References

1. Soo F, Theriot J (2005) Large-scale quantitative analysis of sources of variation in the actin polymerization-based movement of listeria monocytogenes. Biophys J 89: 703–723.

2. Theriot J, Mitchison T (1995) Myosin II filament assemblies in the active lamella of fibroblasts: Their morphogenesis and role in the formation of actin filament bundles. J Cell Biol 131: 989–1002.

3. Jurado C, Haserick J, Lee J (2005) Slipping or gripping? fluorescent speckle microscopy in fish keratocytes reveals two different mechanisms for generating a retrograde flow of actin. Molecular Biology of the Cell 16: 507–518.

4. Forscher P, Smith S (1988) Actions of cytochalasins on the organization of actin filaments and microtubules in a neuronal growth cone. J Cell Biol 107: 1505–1516.

5. Abercrombie M, Heaysman J, Pegrum S (1970) The locomotion of fibroblasts in culture: I. movements of the leading edge. Exp Cell Res 59: 393–398.

6. Dembo M, Wang YL (1999) Stresses at the cell-to-substrate interface during locomotion of fibroblasts. Biophys J 76: 2307–2316.

7. Purcell E (1977) Life at low Reynolds number. Am J of Physics 45(1): 3 –11.

8. Luu N, Rainger G, Buckley C, Nash G (2003) CD31 regulates direction and rate of neutrophil migration over and under endothelial cells. J Vasc Res 40: 467–479.

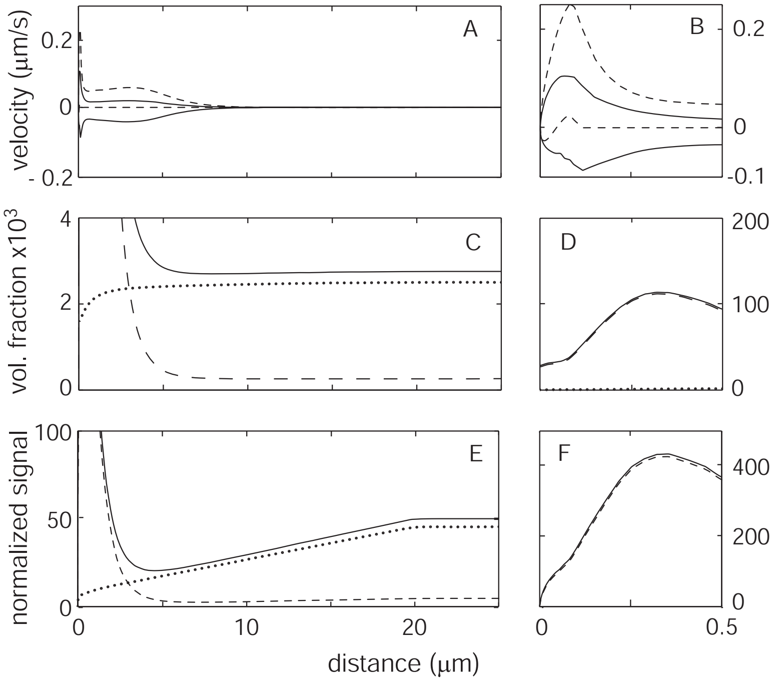

Figure A1. Steady state solutions for Case-2 (high network swelling in end caps). Parameters are as in Case-0, except that the value of the network swelling coefficient in the end caps is increased by a factor of 10. Panels (A) and (B) show the network (*dashed line*) and the solvent (*solid line*) velocities at the top and bottom boundaries. Because conditions in the bulk cytoplasm are unchanged, the dynamics on a large scale are similar to Case-0. Thus the area of strong flow now extends no farther that 10 μm behind the leading edge. There is however a very narrow area near the leading edge where the velocities are high due to the rapid expansion from the front compartment. Panels (C) and (D) show volume fractions of G-, F-, and total actin (*dotted line*, *dashed line*, and *solid line*). Panels (E) and (F) indicate predicted fluorescence intensity normalized to give 50% signal at the cell midpoint (*solid line*). Also shown is the breakdown of the intensity into its G- and F- components (*dotted line* and *dashed line*). The peak intensity at the leading edge is high, but not as high as in Case-0. In addition, the peak of the actin volume fraction is now outside of the leading edge compartment. This means that the location of the off-rate maximum (which coincides with the location of actin peak) is separated from the location of the on-rate maximum (inside the leading edge compartment) by about 0.3 μm.

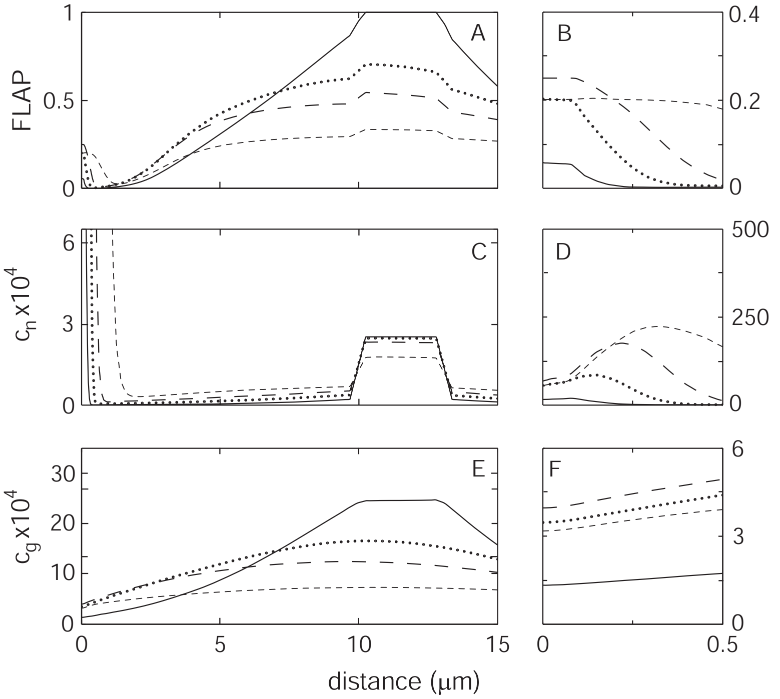

Figure A2. Flap calculation for Case-2 (high network swelling in end caps). Parameters are as in Table 1, except that the value of the network swelling coefficient in the end caps is increased by a factor of 10. Panels (A) and (B) show the spatial distribution of the FLAP signal immediately after the 2-second bleaching period (*solid line*) and at 2, 5, and 15 s (*dotted* line, *long-dash* line, and *short-dash* line). At 2 s, the leading edge value reaches 0.2 - a dramatic increase compared to the Case-0. Panels (C) and (D) show the thickness average of the bleached F-actin volume fraction (c_n). Note from the side panel that the maximum value shifts away from the leading edge with time due to the retrograde flow. Panels (E) and (F) indicate the thickness average of the bleached G-actin volume fraction (c_g). Note that the distribution of labeled G-actin is almost identical to what was seen in Cases-0 and -1.

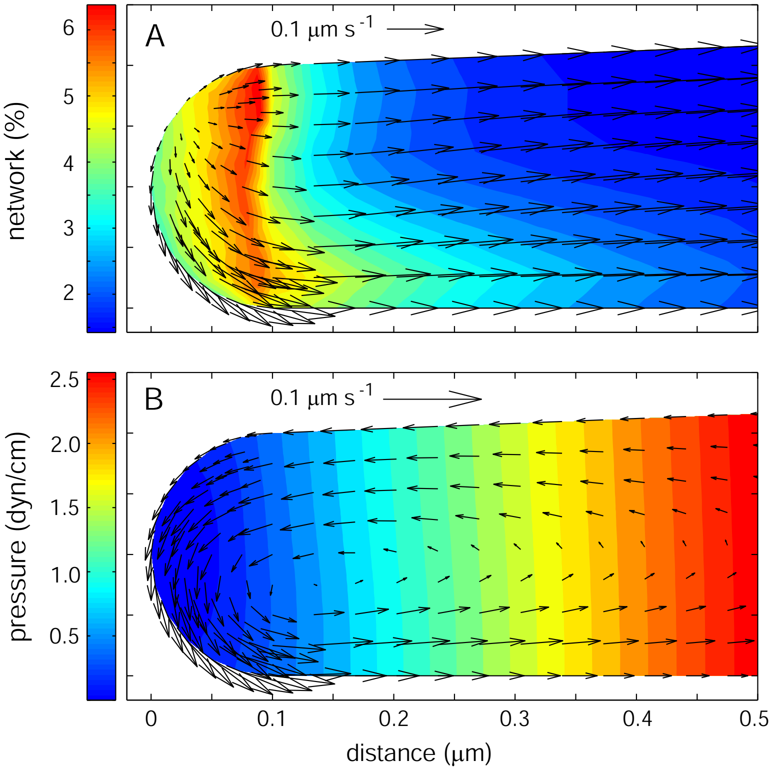

Figure A3. Steady state solutions at the lamella leading edge for Case-3 (gliding cell). The velocities are shown in the frame of reference of the moving cell. Parameters are as in Table 1, except that $\rho_c = \rho_b = 0.1$ in the right side cap. (A) Color contour plot of the network volume fraction with arrows indicating network velocity. The maximum volume fraction is at the dorsal surface inside the cap compartment. The newly created network expands from the leading edge compartment, creating a retrograde flow of polymerized actin towards the main body of the cell. (B) Color contour of the pressure field with solvent velocity indicated by superimposed arrows. The cytosolic flow is entrained with the network along the ventral surface, but is sucked forward by the low pressure of the cap to enforce volume conservation.

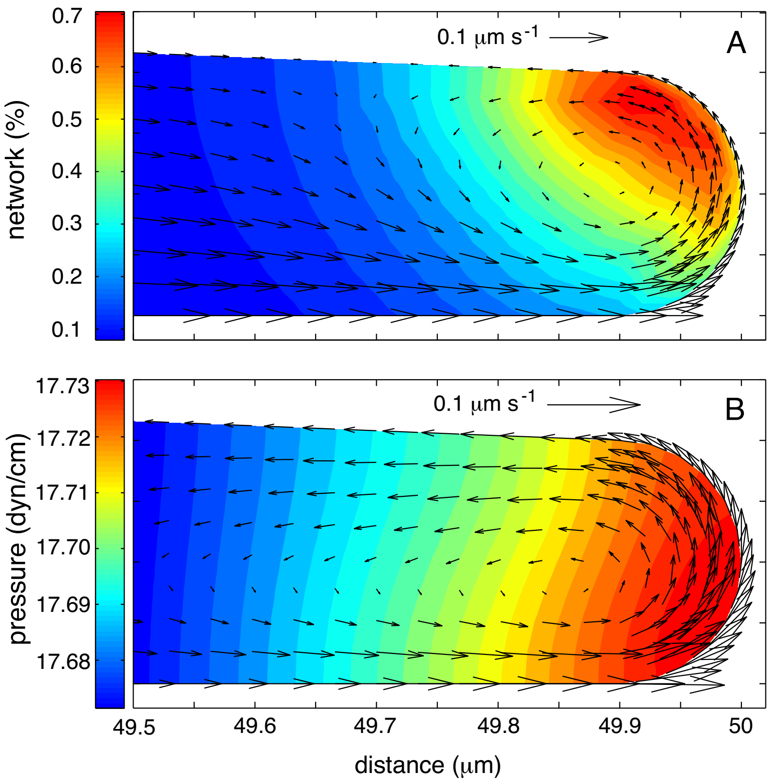

Figure A4. Steady state solutions at the trailing edge for Case-3 (gliding cell). The velocities are shown in the frame of reference of the moving cell. Parameters are as in Table 1, except that $\rho_c = \rho_b = 0.1$ in the right cap. (A) Color contour plot of the network volume fraction with arrows indicating network velocity. The maximum volume fraction is at the dorsal surface inside the cap compartment. The local maximum of F-actin at the trailing edge is due to sweeping of the network by the advancing membrane. (B) Color contour of the pressure field with solvent velocity indicated by superimposed arrows. The cytosolic flow is entrained with the network along the ventral surface, and is pushed out of the trailing edge cap by the elevated pressure so as to maintain constant density.

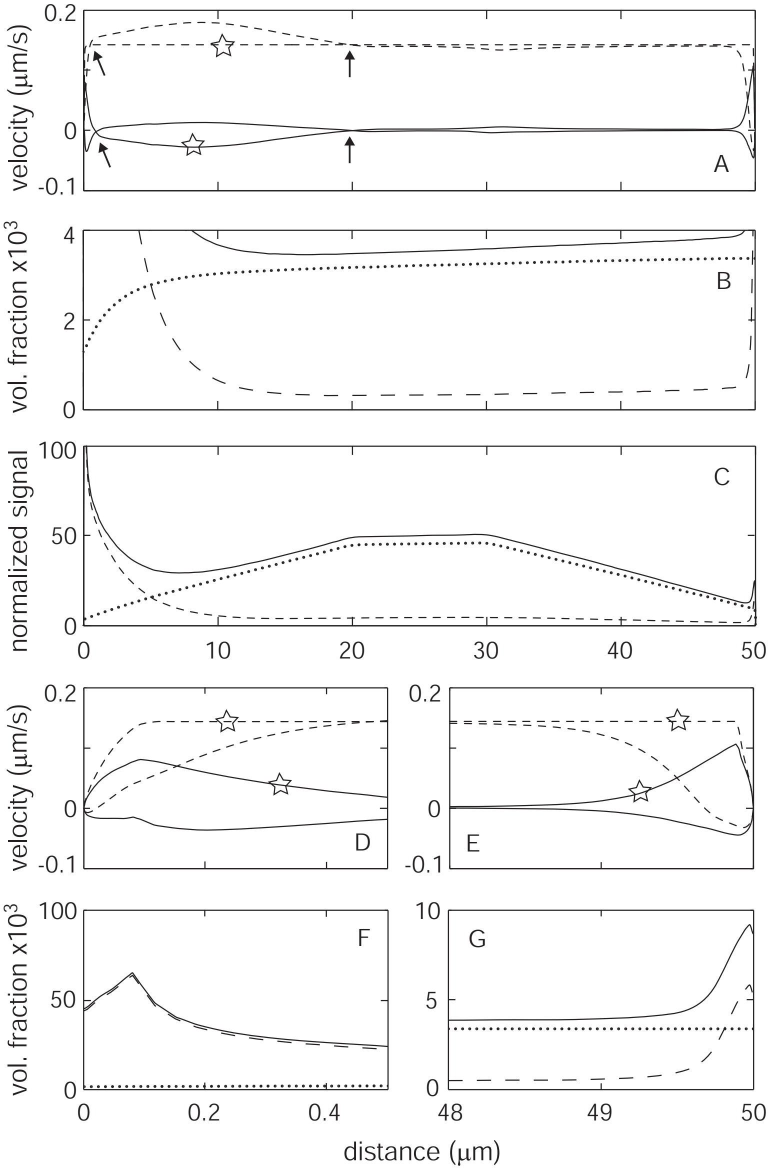

Figure A5. Steady state solutions for Case-3 (gliding cell). Parameters are as in Case-0, except that $\rho_c = \rho_b = 0.1$ in the right hand side cap. Since the left-right symmetry is no longer preserved, the steady state solution is shown for the entire domain. Panels (A), (D), and (E) show the network (*dash line*) and the solvent (*solid line*) velocities at the top and bottom boundaries in the frame of reference of the gliding cell ($U = 0.14\,\mu\mathrm{m\,s}^{-1}$). Star markers indicate the flow at the top boundary; arrows point at the locations where the velocity profiles intersect. Panels (B), (F), and (G) show volume fractions of G-, F-, and total actin (*dotted line*, *dashed line*, and *solid line*). Note that the small local maximum of F-actin at the trailing edge is due to sweeping of the network by the advancing membrane. Panel (C) indicates predicted fluorescence intensity normalized to give 50% signal at the cell midpoint (*solid line*, compare to Fig. 4). Also shown is the breakdown of the intensity into G- and F- components (*dotted line* and *dashed line*).

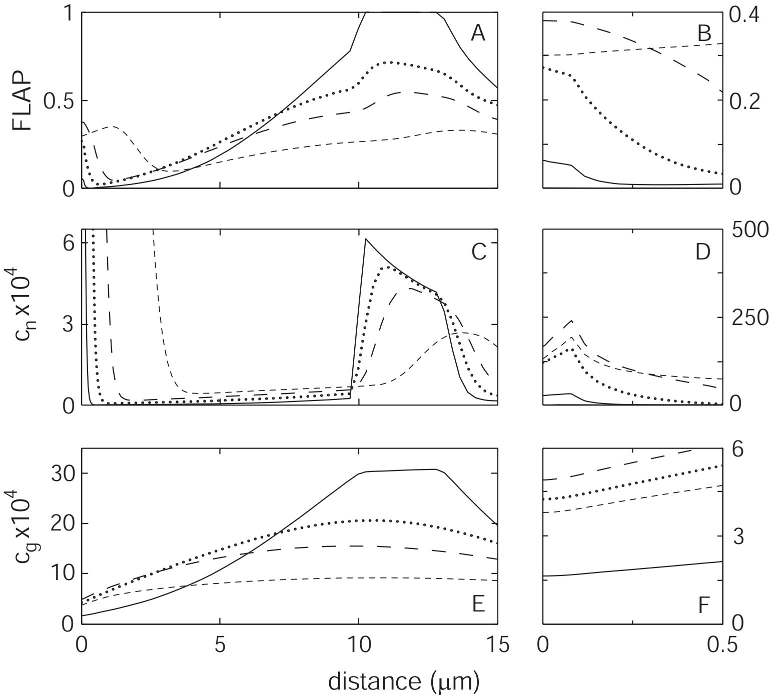

Figure A6. FLAP calculation for Case-3 (gliding cell). Parameters are as in Table 1, except that $\rho_c = \rho_b = 0.1$ in the right hand side cap. Panels (A) and (B) show the spatial distribution of the FLAP signal immediately after the 2 s bleaching period (*solid line*) and at 2, 5, and 15 s (*dotted* line, *long-dash* line, and *short-dash* line). At 2 s, the FLAP value at the leading edge is 0.23, rising to 0.37 at 5 s and declining to the value of 0.30 at 15 s. The drift of the local FLAP maximum from the leading edge towards the cell body seen in the 15 s profile resembles the experimentally observed (Zicha et al.) drift of a similar nature. Panels (C) and (D) show the thickness average of the bleached F-actin volume fraction (c_n). The advection in this case is sufficiently fast so there is an observable transport of the F-actin out of the bleaching zone. Panels (E) and (F) indicate the thickness average of the bleached G-actin volume fraction (c_g).
